# Complementary Transcriptomic and Proteomic Analysis in the Substantia Nigra of Parkinson's Disease

**DOI:** 10.1155/2021/2148820

**Published:** 2021-10-07

**Authors:** Bao-hua Dong, Zhao-qing Niu, Jing-tao Zhang, Yi-jing Zhou, Fan-mei Meng, Ai-qin Dong

**Affiliations:** ^1^Department of Internal Medicine-Neurology, Dongying District People's Hospital of Dongying City, Dongying, Shandong, China; ^2^Department of Respiratory Medicine, Dongying District People's Hospital of Dongying City, Dongying, Shandong, China; ^3^Outpaitient Department, Dongying District People's Hospital of Dongying City, Dongying, Shandong, China

## Abstract

Parkinson's disease (PD) is a disease that involves brain damage and is associated with neuroinflammation, mitochondrial damage, and cell aging. However, the pathogenic mechanism of PD is still unknown. Sequencing data and proteomic data can describe the fluctuation of molecular abundance in diseases at the mRNA level and protein level, respectively. In order to explore new targets in the pathogenesis of PD, the study analyzed molecular changes from the database by combining transcriptomic and proteomic analysis. Differentially expressed genes and differentially abundant proteins were summarized and analyzed. Enrichment and cluster analysis emphasized the importance of neurotransmitter release, mitochondrial damage, and vesicle transport. The molecular network revealed a subnetwork of 9 molecules related to SCNA and TH and revealed hub gene with differential expression at both mRNA and protein levels. It found that ACHE and CADPS could be used as new targets in PD, emphasizing that impaired nerve signal transmission and vesicle transport affect the pathogenesis of PD. Our research emphasized that the joint analysis and verification of transcriptomics and proteomics were devoted to understanding the comprehensive views and mechanism of pathogenesis in PD.

## 1. Introduction

Parkinson's disease (PD) is an age-dependent neurodegenerative disease with the pathological part in the brain. Neurons in the substantia nigra of PD patients are lost massively with lack of striatum dopamine [1]. The damage of dopaminergic neurons is the key to the pathogenesis of PD, which is accompanied by cell aging, mitochondrial damage, and inflammation [2]. PTEN-induced putative kinase protein 1 (PINK1) and parkin (PARK2) were a typical mitochondrial phagocytic pathway protein, which is related to the ubiquitination of mitochondrial outer membrane proteins and the degradation of mitochondria [3, 4]. Chronic inflammation and continuous activation of microglia can lead to excessive release of ROS, which in turn leads to nerve inflammation. The superoxide dismutase 1 (SOD1) and superoxide dismutase 2 (SOD2) are involved in the occurrence of neuroinflammation [5]. However, the pathogenesis and new effective molecular targets are not clear.

RNA sequencing has been reported in the pathogenesis, and quantitative proteomics plays a role in the pathogenesis of PD, providing molecular abundance change in PD patient tissues [6, 7]. Blood sequencing of patients with early Parkinson's disease was used to screen for specific markers to improve the effectiveness of clinical diagnosis [8]. Core genes, such as PSMA4, PSMB1, PSMC5, and PSME4, and pathways, were constructed to improve understanding of the molecular mechanisms of Parkinson's disease [9, 10].

On the other hand, Proteomics, a high throughput technique based on mass spectrometry, has shown superiority in revealing the pathogenesis of Parkinson's disease, and the proteomics of the substantia nigra has provided new insights to explain the prominent dysfunction in PD patients [11, 12]. It is of great convenience and efficiency that the genetic microarrays illustrated the crossreactivity of PD patient tissues and probes, and the mass spectrometry-based proteomics could be used to analyze the changes in protein abundance fluctuations in substantia nigra of PD [13]. This study explored the molecular changes of the pathogenic process by focusing on sequencing data and quantitative proteomics in the substantia nigra tissue of PD patients.

## 2. Materials and Methods

### 2.1. Identification of Differential Expression in the Substantia Nigra of PD

The data of the substantia nigra region of Parkinson's patients were extracted from the literature, and only the transcriptome and proteome data of the substantia nigra region were considered. In addition, the sequencing data of substantia nigra in PD patients was extracted from GEO Datasets (https://http://www.ncbi.nlm.nih.gov/gds/). Proteomic results in the substantia nigra of PD patients are sourced from the proteomeXchange database (http://www.proteomexchange.org/). Pearson analysis calculates the *r* value by fitting the correlation curve. The threshold of fold change was set to 2 (∣FC | ≥2). Significant differences were described by calculating *p* values (*p* < 0.05).

### 2.2. Functional Enrichment Analysis

Gene ontology (GO) analysis was used for clustering and enrichment analysis. Pathway analysis was described by Kyoto Encyclopedia of Genes and Genomes (KEGG). The Database for Annotation, Visualization, and Integrated Discovery (DAVID, version 6.8) [14] was used for enrichment analysis including biological process, molecular function, and cell composition and KEGG analysis (https://david.ncifcrf.gov/tools.jsp).

### 2.3. GO Chord Diagram and Correlation Analysis

Visualization of GO analysis was performed through GO chord diagrams to show the relationship between molecular expression and enrichment items. KEGG bubble maps were used to map the results of pathway analysis using bioinformatic tools (http://www.bioinformatics.com.cn). Pearson's analysis was used to reveal correlations between transcriptomic and proteomic data, and Pearson's coefficients *r* were used to indicate positive or negative correlations.

### 2.4. Construction of Interaction Networks

Molecular networks are based on interactions including textmining, experiments, databases, coexpression, neighborhood, gene fusion, and co-occurrence. The molecular network was constructed by STRING (https://string-db.org/cgi/input.pl), and the social network was drawn and optimized by Cytoscape software (version 3.7.2) [15]. MCODE, a plug-in of Cytoscape, was used to analyze and prompt subnetworks and scoring.

### 2.5. Statistical Analysis

Visualization and analysis of data were performed in Prism (version 8.0, GraphPad, San Diego, CA, USA). Student's *t*-test was used to analyze the significance of the data, and values of *p* < 0.05 were set statistically significant and ^∗^*p* < 0.05, ^∗∗^*p* < 0.01, ^∗∗∗^*p* < 0.001.

## 3. Results

### 3.1. Expression Profile in the Substantia Nigra of PD

**Figure 1:**
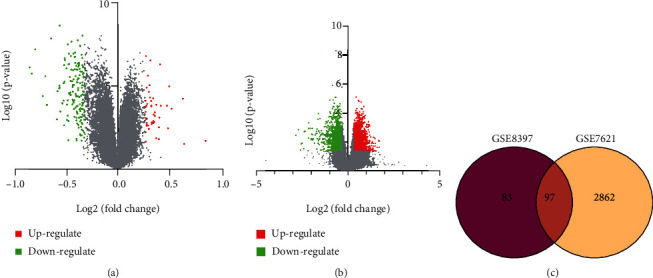
Transcriptomic gene expression and enrichment analysis in GSE8397 and GSE7621. (a) Differential expression analysis of genes in GSE8397. (b) Volcano graphs of GSE7621. (c) Venn diagram of differentially expressed in GSE8397 and GSE7621.

Analysis of transcriptomics is an effective method to describe changes in the gene expression during the occurrence and development of diseases [16]. To understand the expression of mRNA levels in the focal area, we compared the substantia nigra microarray data of PD patients and healthy control. Focusing on the genetic factors of complex diseases, we compiled the research of Duke et al. [17, 18] and Lesnick et al. [19] on DNA oligonucleotide microarrays to describe a comprehensive gene expression profile. The gene expression in the substantia nigra of PD patients is fully described in independent GEO datasets including GSE8397 and GSE7621 [18, 19]. There are 72 samples, including 45 PD patients and 27 healthy controls. All data in GSE8397 (GPL97), GSE8397 (GPL96), and GSE7621 (GPL570) have been normalized and can be crosscompared in differential expression studies. A total of 186 differentially expressed genes (DEGs) were observed in GSE8397, of which 39 were upregulated and 147 were downregulated (Figure 1(a)). And 2955 differentially expressed genes (DEGs) were observed in GSE7621, of which 1657 were upregulated and 1298 were downregulated (Figure 1(b)). The drawing of the Venn diagram revealed that a total of 96 molecules were differentially expressed in the two datasets (Figure 1(c)).

### 3.2. Clustering and Enrichment Analysis of DEGs

**Figure 2:**
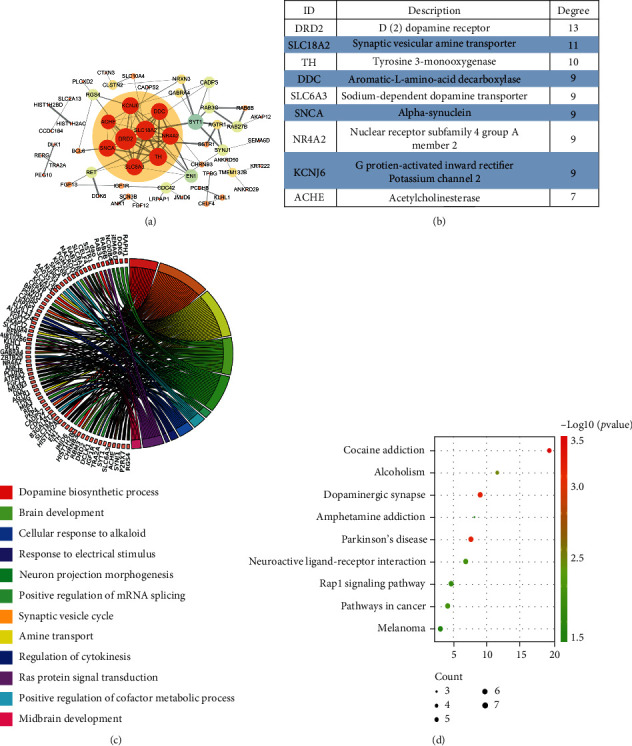
Molecular network construction and cluster analysis of 96 DEGs in GSE8397 and GSE7621. (a) The interaction network of 96 molecules. (b) A subnetwork was mined by MCODE. (c) Enriched chord diagrams are used to describe GO analysis of 96 molecules. (d) The bubble chart of the pathway analysis was enumerated by using KEGG analysis.

To understand the interactions of DEGs, we constructed a social network. The interaction network of 96 molecules was enriched in STRING and artisticized in Cytoscape (Supplemental Table S1). A subnetwork containing DRD2, SLC18A2, TH, DDC, SLC6A3, SNCA, NR4A2, KCNJ6, and ACHE molecules was mined by MCODE (Figures 2(a) and 2(b)). SNCA and TH, the highly correlated protein of PD, occupied the center of the subset, which indirectly implied the potentially important role in PD.

To explore the physiological functions macroscopically of DEGs, we conducted the clustering enrichment analysis. GO enrichment analysis revealed that 96 molecules participated in dopamine biosynthetic process, synaptic vesicle cycle, amine transport, brain development, neuron projection morphogenesis, positive regulation of mRNA splicing, via spliceosome, positive regulation of cofactor metabolic process, cellular response to alkaloid, regulation of cytokinesis, response to electrical stimulus, Ras protein signal transduction, and midbrain development (Figure 2(c)). Signaling pathways including cocaine addiction, ampheamine addiction, dopaminergic synapse, melanoma, alcoholism, Parkinson's disease, Rap1 signaling pathway, neuroactive ligand-receptor interaction, and pathways in cancer were observed in KEGG analysis (Figure 2(d)).

**Figure 3:**
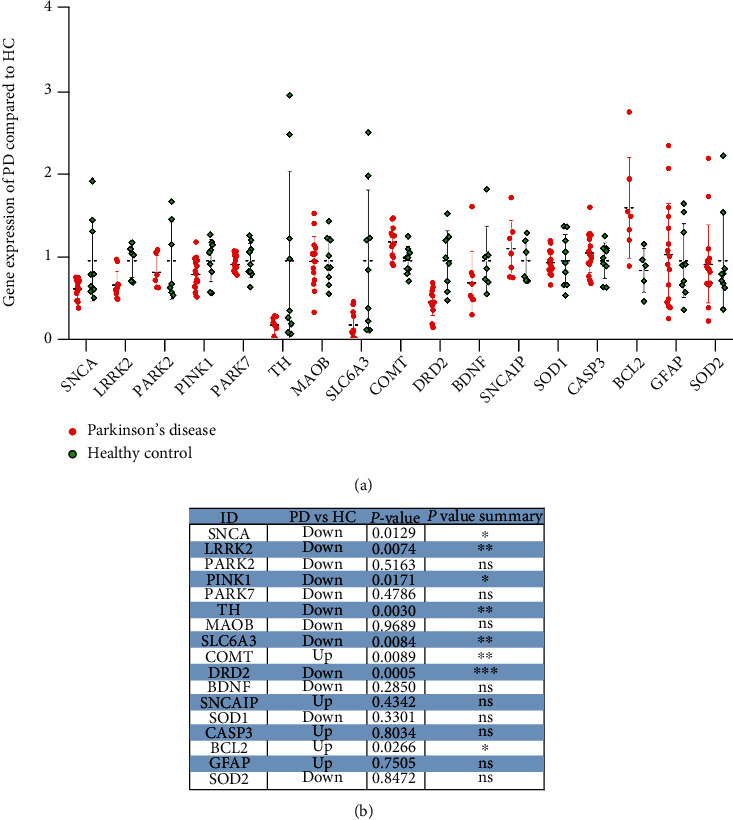
Differential expression and significance in DEGs of hub genes of PD. (a) The expression of genes related to PD with high scores was extracted including SNCA, LRRK2, PINK1, TH, BDNF, SOD2, COMT, SNCAIP, CASP3, GFAP, and BCL2. (b) The hub genes of PD were counted for differential expression and significance.

To analyze the related-molecules of PD, the Digsee database (http://210.107.182.61/geneSearch/) was used to summarize the known targets of disease, and 516 PD-related molecules were retrieved. The expression of genes related to PD with high scores was extracted and found that SNCA, LRRK2, PINK1, TH, BDNF, and SOD2 were downregulated, while COMT, SNCAIP, CASP3, GFAP, and BCL2 were upregulated (Figures 3(a) and 3(b)).

### 3.3. Combined Transcriptomic and Proteomic Analysis of Substantia Nigra

**Figure 4:**
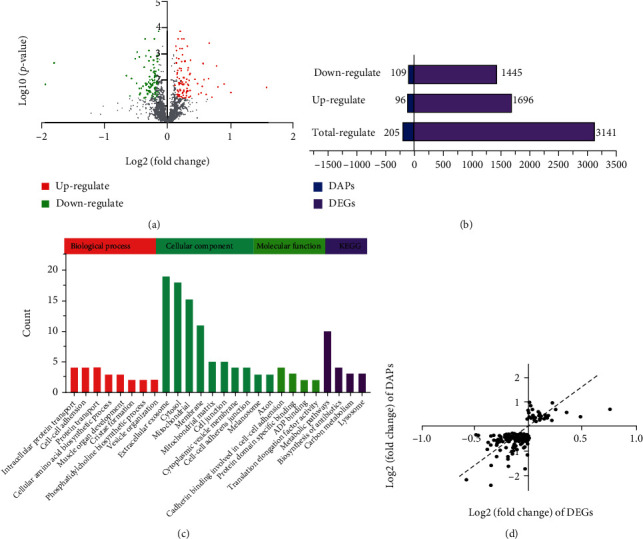
Comparative analysis of transcriptomics and proteomics in SN of PD. (a) Protein expression of DAPs in volcano map of PXD000427. (b) DEGs and DAPs in GSE8397, GSE7621, and PXD000427 were listed. (c) The 44 molecules differentially expressed in both transcriptomics and proteomics were analyzed by GO enrichment analysis. (d) Pearson correlation analysis of mRNA and protein expression.

In order to explore the protein fluctuations in the process of disease, we analyzed the proteomics profile of PD patients. Licker and colleagues [20] performed a proteomic analysis of the substantia nigra region of PD patients, and the PXD000427 dataset was obtained in the proteomeXchange database, including 5 PD patients and 8 controls. The overall expression of the detected protein was described by the volcano graph (Figure 4(a)). Proteomic results showed that a total of 205 differential abundance proteins (DAPs) were observed, of which 96 were upregulated and 109 were downregulated (Figure 4(b)).

In order to explore and discover new pathogenic mechanisms in the pathogenesis of PD, we jointly interpreted transcriptomics and proteomics. 44 molecules were both differentially expressed in profiles' transcriptomics and proteomics (Supplemental Table S2). The 44 molecules were enriched and analyzed by KEGG and GO analysis (Figure 4(c)). GO enrichment analysis found that DAPs were localized to the membrane, played the molecular function of binding, and participated in the physiological functions of transport and biosynthetic process. Biological process analysis showed that 44 molecules were significantly involved in nerve synapses and vesicles, including transsynaptic signaling (GO :0099537), synaptic signaling (GO: 0099536), chemical synaptic transmission (GO: 0007268), anterograde transsynaptic signaling (GO: 0098916), modulation of synaptic transmission (GO: 0050804), phosphatidylcholine biosynthetic process (GO: 0006656), vesicle organization (GO: 0016050), vesicle-mediated transport (GO: 0016192), and intracellular protein transport (GO: 0006886). Pearson's analysis was used to analyze the correlation of DEGs and DAPs molecules (Figure 4(d)). The *r* coefficient was positive, and the *R*^2^ value is 0.4566, which indicated that there was a positive correlation between transcriptomics and proteomics.

### 3.4. Network Analysis of Differentially Expressed Molecules

**Figure 5:**
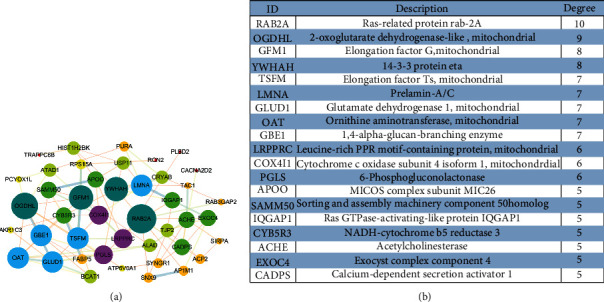
Network analysis of 44 hub genes in both transcriptomics and proteomics. (a) The 44 molecules differentially expressed in both transcriptomics and proteomics were analyzed by STRING. (b) Hub genes with high degrees were listed and described.

To explore the potential key molecular targets in the pathogenesis of PD, we carried out the construction of molecular networks. Interaction networks were used to evaluate molecules at hub nodes in DEGs and DAPs (Figure 5(a)). All 44 molecules have been connected by Cytoscape, and the key molecules have been sorted according to degree including RAB2A, OGDHL, GFM1, YWHAH, TSFM, LMNA, GLUD1, OAT, GBE1, LRPPRC, COX4I1 PGLS, APOO, SAMM50, IQGAP1, CYB5R3, ACHE, EXOC4, and CADPS (Figure 5(b)). The highly socialized molecules suggested that they had strong correlations and can be new target proteins.

### 3.5. Verification That ACHE and CADPS Can Be Used as New Target Molecules of PD

**Figure 6:**
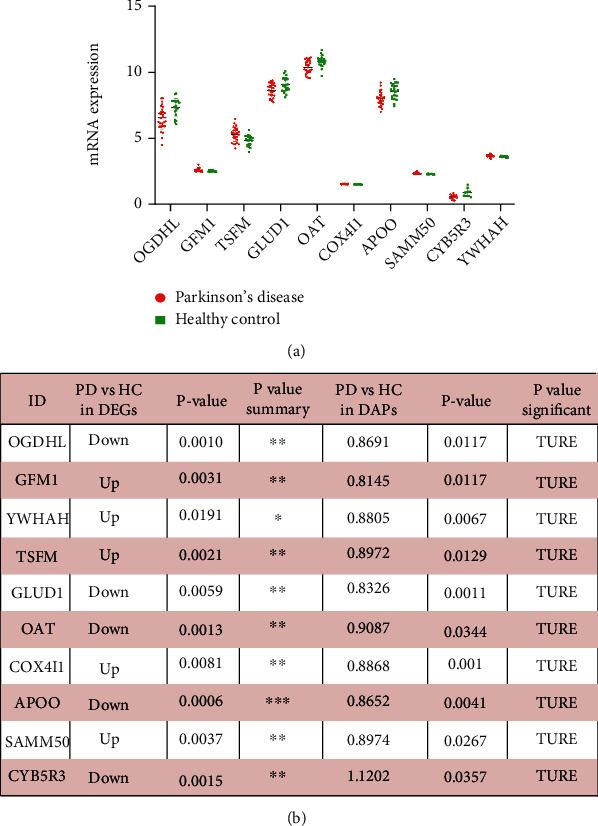
The abundance of mitochondrial molecules expression in PD patients. (a) The mRNA expression of mitochondrial molecules in hub gene. (b) The mitochondrial molecules in the hub gene were counted and analyzed for mRNA expression and relative protein abundance.

Mitochondrial proteins showed high scores and a strong correlation in PD patients. Studies have shown that mitochondria are becoming a new therapeutic target in PD [21]. Mitochondria-targeted agents can afford neuroprotection in preclinical mice of PD. So, we speculate that mitochondrial proteins can become new therapeutic targets including OGDHL, GFM1, YWHAH, TSFM, GLUD1, OAT, COX4I1, APOO, SAMM50, and CYB5R3 (Figure 6(a)). They showed significant differential expression in both transcriptomics and proteomics (Figure 6(b)).

**Figure 7:**
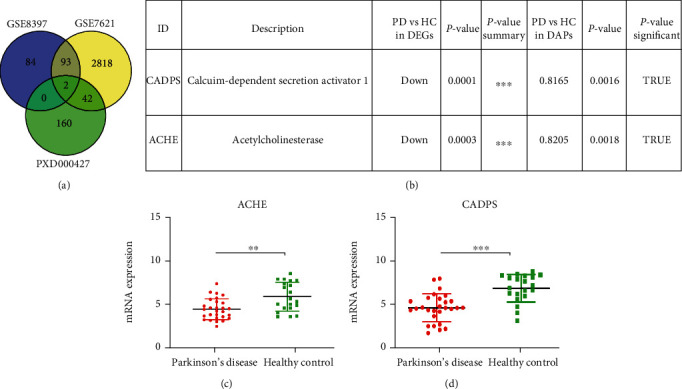
ACHE and CADPS can be used as new target molecules of PD. (a) The Venn diagram was used to describe the relationship between transcriptomics and proteomics. (b) ACHE and CADPS have been described and analyzed the expression abundance in transcriptomics and proteomics. (c) The mRNA expression level of ACHE in GSE8397 and GSE7621. (d) The mRNA expression level of CADPS in GSE8397 and GSE7621. ^∗∗^*p* < 0.01, ^∗∗∗^*p* < 0.001.

In order to explore new target molecules, we aggregated differentiated molecules through combined transcriptomics and proteomic analysis. It was found that the two molecules showed significant differential expression at both the mRNA and the protein expression level (Figure 7(a)). ACHE, acetylcholinesterase, is involved in the process of neurotransmitter hydrolysis and the termination of nerve signal transmission [22]. CADPS, calcium-dependent secretion activator [23], encodes a calmodulin-related protein and regulates the process of vesicle exocytosis (Figure 7(b)). ACHE and CADPS were verified to be significantly downregulated (Figures 7(c) and 7(d)). It showed that the impairment of neurotransmitter transmission and vesicle transport played a critical role in the pathogenesis of PD.

## 4. Discussion

Transcriptomics and proteomics were used jointly to explain the pathogenesis of PD at the mRNA and protein levels [24]. The advantage of transcriptomics lies in the ease of microarray or library recognized RNA expression through bulky probes, but real and useful data were hidden in individual differences, which was difficult to eliminate. It was well known that the impact of environmental toxins was also one of the pathogenesis of PD, but it could not be reflected in the sequencing results. The method, exploring molecular changes in protein and protein modification in disease development, is to comprehensively elaborate the study of molecular changes and describe the mechanism, and it was named proteogenomic [25].

The pathological physiological process was composed of a complex molecular network including SNCA, LRRK2, PINK1, TH, BDNF, BCL2, CASP3, GFAP, and SOD2, which involved neuroinflammation, mitochondrial damage, apoptosis, and neurotrophic. A subnetwork may become a remote controller for complex regulation of DA secretion. Lewy bodies, formed by the accumulation of *α*-synuclein, were observed in the substantia nigra area of PD patients [26]. The loss of TH neurons was one of the hallmarks of PD. Therefore, the subnetwork was considered to be an important subset of PD pathogens including DRD2, SLC18A2, TH, DDC, SLC6A3, SNCA, NR4A2, KCNJ6, and ACHE, and they were involved in molecular function of protein binding with signaling pathways including carbon metabolism, metabolic pathway, and biosynthesis of antibiotics.

RAB2A was an essential part of endoplasmic reticulum to Golgi transport, encoded membrane-bound protein Rab, and participated in vesicle fusion and transport. RAB2A propagated functional autophagy signals through binding to the ULK1 complex [27, 28], but the function of RAB2A in the pathogenesis of PD remained elusive. COX4I1 (cytochrome c oxidase subunit 4I1) was a mitochondria-localized gene, which is related to mitochondrial fusion and fission in PD [29, 30]. Pathogenic GFM1 caused damage to basal ganglia, brainstem, and periventricular white matter. Mitochondrial elongation factor G1 (GFM1) was involved in oxidative phosphorylation and energy transfer processes [31, 32], but the function of GFM1 in PD remained unclear.

ACHE and CADPS were screened and verified to be downregulated in PD patients. ACHE was able to form various acetylcholinesterases (ACHE) through mRNA splicing and protein translation. ACHE existed on the cell surface as an oligomer. ACHE had catalytic activity and could hydrolyze neurotransmitters, acetylcholine ,and brain choline to terminate signaling. ACHE in the cerebral cortex has been found to be upregulated in Parkinson's dementia, which can influence the cognitive function [33]. CADPS encoded neural-specific cytosolic membrane proteins and participated in Ca2+ regulated vesicle trafficking. The downregulation of CADPS and the weakening of the secretion of CADPS protein were involved in the neuroprotective role in the progression of PD [34, 35].

## 5. Conclusion

Our research emphasizes that the joint analysis and verification of transcriptomics and proteomics were devoted to understanding the comprehensive views and mechanism of pathogenesis in PD.

## Data Availability

All available data are downloaded from GEO (https://www.ncbi.nlm.nih.gov/gds).

## Supplementary Materials

Supplementary 1 Table S1: the interaction network of 96 molecules was enriched in STRING and artisticized in Cytoscape.

Supplementary 2 Table S2: 44 molecules were both differentially expressed in profiles' transcriptomics and proteomics.
