# Supplementary material for: Complementary Transcriptomic and Proteomic Analysis in the Substantia Nigra of Parkinson's Disease
Source: Dis Markers. 2021 Oct 7;2021:2148820. doi: 10.1155/2021/2148820 (PMC8517625; doi:10.1155/2021/2148820)
Supplement: Supplementary 1 — Table S1: the interaction network of 96 molecules was enriched in STRING and artisticized in Cytoscape. [file 2148820.f1.pdf]

Table S1: The interaction network of 96 molecules was enriched in STRING and artisticized in Cytoscape.

RAPH1
DOK6
PLCXD2
SEMA6D
LINC00849
RAB6B
RAB3C
dao
SSTR1
ANKRD29
CELF4
CCDC184
C2orf80
AI590238
SLC8A1
RAB27B
CTXN3
ANKRD50
MACROD2
PGM2L1
BEND4
TMEM178B
KIF26B
KRT222
SNCA
NUP93
AA035730
SLC2A13
KCNJ6
HENMT1
CEP41
TMEM132B
BRWD1
PSMA5
EBF3

AW296451
FSD1L
AA832474
CADPS
LRPAP1
KLHL13
AL041224
FGF12
TIMM23B
AKAP12
SLC10A4
RERG
MIR612
GTF3A
N71087
AI810266
KLHL1
BCL6
WDR37
GABRA4
C7orf61
ZBTB20
NR4A2
ANK1
PCDH8
ATP8A2
FGF13
NRXN3
DLK1
DDC
AGTR1
TPBG
MIA3
RET
SCN3B
ZNF226
PKP4
CADPS2

CDC42
B3GALNT1
SLC18A2
HIST1H2AC
EN1
TH
JMJD6
HIST1H2BD
CYB561
CHRNB3
RBM3
PEG10
DRD2
DCLK1
IGF1R
TRA2A
SYT1
SLC6A3
ACHE
SYNJ1
P2RX7
RGS4
TMEM255A
