# Supplementary material for: Complementary Transcriptomic and Proteomic Analysis in the Substantia Nigra of Parkinson's Disease
Source: Dis Markers. 2021 Oct 7;2021:2148820. doi: 10.1155/2021/2148820 (PMC8517625; doi:10.1155/2021/2148820)
Supplement: Supplementary 2 — Table S2: 44 molecules were both differentially expressed in profiles' transcriptomics and proteomics. [file 2148820.f2.pdf]

Table S2: 44 molecules were both differentially expressed in profiles transcriptomics and proteomics.

ID	Ratio	Significant
ACHE	0.8205	TRUE
ACP2	0.805	TRUE
AKR1C3	1.4898	TRUE
ALAD	1.2783	TRUE
AP1M1	0.9048	TRUE
APOO	0.8652	TRUE
ATAD1	0.8607	TRUE
ATP6V0A1	0.8687	TRUE
BCAT1	0.7865	TRUE
CACNA2D2	0.8509	TRUE
CADPS	0.8165	TRUE
COX4I1	0.8868	TRUE
CRYAB	1.1761	TRUE
CYB5R3	1.1202	TRUE
EXOC4	0.8901	TRUE
FABP5	1.1056	TRUE
FMNL2	1.1123	TRUE
GBE1	0.7878	TRUE
GFM1	0.8145	TRUE
GLUD1	0.8326	TRUE
HIST1H2BK	1.2837	TRUE
IQGAP1	1.168	TRUE
LMNA	1.1159	TRUE
LRPPRC	0.7878	TRUE
OAT	0.9087	TRUE
OGDHL	0.8691	TRUE
PCYOX1L	0.7751	TRUE
PGLS	1.1949	TRUE
PLBD2	0.8452	TRUE
PURA	0.7506	TRUE
RAB2A	0.8985	TRUE
RAB3GAP2	0.8343	TRUE
RCN2	0.864	TRUE
RPS15A	0.8676	TRUE
SAMM50	0.8974	TRUE
SIRPA	1.1749	TRUE
SNX9	1.1198	TRUE
SYNGR1	0.8547	TRUE
TAC1	0.8405	TRUE
TJP2	1.7103	TRUE
TRAPPC6B	0.8693	TRUE

TSFM	0.8972	TRUE
USP11	0.851	TRUE
YWHAH	0.8805	TRUE
